# Whole-genome sequencing and comparative genomic analyses of the medicinal fungus *Sanguinoderma infundibulare* in Ganodermataceae

**DOI:** 10.1093/g3journal/jkae005

**Published:** 2024-02-15

**Authors:** Yuxuan Fang, Dongmei Wu, Neng Gao, Mengxue Lv, Miao Zhou, Chuangui Ma, Yifei Sun, Baokai Cui

**Affiliations:** State Key Laboratory of Efficient Production of Forest Resources, School of Ecology and Nature Conservation, Beijing Forestry University, Beijing 100083, China; Xinjiang Production and Construction Group Key Laboratory of Crop Germplasm Enhancement and Gene Resources Utilization, Biotechnology Research Institute, Xinjiang Academy of Agricultural and Reclamation Sciences, Shihezi 832061, China; Xinjiang Production and Construction Group Key Laboratory of Crop Germplasm Enhancement and Gene Resources Utilization, Biotechnology Research Institute, Xinjiang Academy of Agricultural and Reclamation Sciences, Shihezi 832061, China; State Key Laboratory of Efficient Production of Forest Resources, School of Ecology and Nature Conservation, Beijing Forestry University, Beijing 100083, China; State Key Laboratory of Efficient Production of Forest Resources, School of Ecology and Nature Conservation, Beijing Forestry University, Beijing 100083, China; Beijing Jingcheng Biotechnology Co., Ltd, Beijing 100083, China; State Key Laboratory of Efficient Production of Forest Resources, School of Ecology and Nature Conservation, Beijing Forestry University, Beijing 100083, China; State Key Laboratory of Efficient Production of Forest Resources, School of Ecology and Nature Conservation, Beijing Forestry University, Beijing 100083, China

**Keywords:** *Sanguinoderma*, Ganodermataceae, medicinal fungi, comparative genomic analyses, next-generation sequencing

## Abstract

*Sanguinoderma infundibulare* is a newly discovered species of Ganodermataceae known to have high medicinal and ecological values. In this study, the whole-genome sequencing and comparative genomic analyses were conducted to further understand Ganodermataceae's genomic structural and functional characteristics. Using the Illumina NovaSeq and PacBio Sequel platforms, 88 scaffolds were assembled to obtain a 48.99-Mb high-quality genome of *S. infundibulare*. A total of 14,146 protein-coding genes were annotated in the whole genome, with 98.6% of complete benchmarking universal single-copy orthologs (BUSCO) scores. Comparative genomic analyses were conducted among *S. infundibulare*, *Sanguinoderma rugosum*, *Ganoderma lucidum*, and *Ganoderma sinense* to determine their intergeneric differences. The 4 species were found to share 4,011 orthogroups, and 24 specific gene families were detected in the genus *Sanguinoderma*. The gene families associated with carbohydrate esterase in *S. infundibulare* were significantly abundant, which was reported to be involved in hemicellulose degradation. One specific gene family in *Sanguinoderma* was annotated with siroheme synthase, which may be related to the typical characteristics of fresh pore surface changing to blood red when bruised. This study enriched the available genome data for the genus *Sanguinoderma*, elucidated the differences between *Ganoderma* and *Sanguinoderma*, and provided insights into the characteristics of the genome structure and function of *S. infundibulare*.

## Introduction

Ganodermataceae is an important family of macrofungi with high economic and ecological values. Recently, its species diversity, taxonomic system, and molecular phylogeny have been extensively studied (Sun *et al*. 2020; Sun, Xing, *et al.* 2022). The genus *Sanguinoderma* Y.F. Sun, D.H. Costa, & B.K. Cui was established in 2020 belonging to Ganodermataceae, and its species are characterized by the color of their fresh pore surface changing to blood red when bruised (Sun *et al*. 2020). Currently, 18 species have been included in the genus *Sanguinoderma* worldwide (Sun, Lebreton, *et al.* 2022). Among them, *Sanguinoderma infundibulare* B.K. Cui & Y.F. Sun is distinctive due to its annual basidiomata with thin and funnel-shaped pileus (Sun *et al*. 2020).

*Sanguinoderma* species have been found to contain a large number of secondary metabolites, such as polysaccharides, sterols and unsaturated fatty acids, that are extremely effective against tumor progression (Shiu *et al*. 2022), cardiovascular disease (Li *et al*. 2022), inflammation (Mai *et al*. 2022), antiproliferative activities (Chen, Zhang, *et al.* 2021), and many other disorders (Zheng *et al*. 2022). In addition, Ganodermataceae species can also secrete enzymes to degrade lignin and cellulose, playing important roles in wood decomposition and bleaching, and can be used for environmental protection and economic applications (Zhou *et al*. 2013). The laccase enzyme in *Ganoderma lucidum* Pat. was found to have excellent effects on the degradation of chlorine strife (Deng *et al*. 2022) and polycyclic aromatic hydrocarbons (Agrawal *et al*. 2018). However, there are no relevant studies on the degradation ability of *Sanguinoderma* species.

Whole-genome sequencing has been completed for an increasing number of Ganodermataceae species, which contributes to a deeper understanding of their biological and genetic characteristics from the genomic perspective. In Ganodermataceae, *G. lucidum* was the first species to be subjected to whole-genome sequencing on Roche 454 and Illumina platforms. The study specifically investigated the synergistic role of cytochrome P450s (CYPs), transporters, and regulatory proteins in secondary metabolism (Chen *et al*. 2012). Owing to the extensive genomic studies conducted on *G. lucidum*, this species has become a model organism for the investigation of secondary metabolic pathways and their regulation in medicinal fungi. The whole-genome sequencing, DNA methylation patterns, and small RNA transcriptome analyses of *Ganoderma sinense* J.D. Zhao, L.W. Hsu, & X.Q. Zhang were conducted to investigate mainly secondary metabolism and genome defense processes (Zhu *et al*. 2015). *Ganoderma leucocontextum* T.H. Li, W.Q. Deng, Sheng H. Wu, Dong M. Wang, & H.P. Hu, *Ganoderma australe* Pat., *Ganoderma boninense* Pat., and *Ganoderma lingzhi* Sheng H. Wu, Y. Cao, & Y.C. Dai were also sequenced to further explore the secondary metabolic synthesis pathways and its related genes in the Ganodermataceae (Utomo *et al*. 2018; Agudelo-Valencia *et al*. 2020; Liu *et al*. 2021; Sun, Fang, *et al.* 2022). However, most genomic studies focused on *Ganoderma* species, and currently only *Sanguinoderma rugosum* Y.F. Sun, D.H. Costa, & B.K. Cui has been sequenced and annotated (Lin *et al*. 2021). The sequencing results suggested that *S. rugosum* has a large number of gene clusters related to carbohydrate-active enzymes and biosynthetic secondary metabolites, laying a foundation for the further understanding of the genomic differences between the genera *Sanguinoderma* and *Ganoderma*.

In this study, next-generation sequencing and third-generation sequencing were combined to obtain high-quality whole-genome data for *S. infundibulare.* The protein-coding genes of *S. infundibulare* were annotated based on several functional databases. Comparative genomic analyses were conducted among *S. infundibulare*, *S. rugosum*, *G. lucidum*, and *G. sinense.* This study enriched the available genome data for *Sanguinoderma* species, and it also contributes to the analyses and utilization of secondary metabolites produced by Ganodermataceae species.

## Materials and methods

### Strains and DNA extraction

Fruiting bodies of *S. infundibulare* Cui 17238 were collected from Guangdong Province, southern China. The dikaryotic strain was isolated directly from the fruiting bodies, and it was deposited at the Institute of Microbiology, Beijing Forestry University. It is available upon request. The strain used for whole-genome sequencing was identified following Sun, Xing, *et al.* (2022). The *S. infundibulare* strain Cui 17238 was inoculated in liquid medium (malt extract medium, 2% malt powder, 1% glucose, and 0.3% KH_2_PO_4_), and cultured in a shake flask at 26°C and 150 rpm/min for 7 days. Then, the mycelia were collected by filtration under aseptic conditions and snap frozen in liquid nitrogen. Genomic DNA extraction using the modified cetyltrimethylammonium bromide extraction method and RNA was extracted by Magnetic Tissue Total RNA Kit (Tiangen, Beijing, China).

### Genome sequencing and assembly

The standard Illumina TruSeq Nano DNA LT library preparation procedure (Illumina TruSeq DNA Sample Preparation Guide) and the TruSeqTM DNA Sample Prep Kit (Illumina, San Diego, USA) were used to construct the genomic upload library for next-generation sequencing, while the standard PacBio Template Prep Kit 1.0 library preparation procedure (20-kb Template Preparation Using BluePippin Size Selection) and the SMRTbellTM Template Prep Kit 1.0 (Pacific Biosciences, California, USA) were used to construct the genomic upload library for third-generation sequencing.

Before sequencing, the libraries were quality checked on an Agilent Bioanalyzer using the Agilent High Sensitivity DNA Kit (Agilent, California, USA). After passing the quality check, the libraries were quantified using the Quant-iT PicoGreen dsDNA Assay Kit (Thermo Fisher, Waltham, USA) on a Promega QuantiFluor fluorescence quantification system.

The whole-genome shotgun strategy was adopted separately using next-generation sequencing technology on an Illumina NovaSeq (Illumina, San Diego, USA) sequencing platform and using third-generation single-molecule sequencing technology on a PacBio Sequel (Pacific Biosciences, California, USA) sequencing platform.

Further filtering of downstream data was required to ensure the quality of subsequent data analyses. Joint contamination was removed from the 3′ end of raw data using AdapterRemoval v2 (Schubert *et al*. 2016), and all reads were quality corrected based on K-mer frequencies using SOAPec v2.0 (Luo *et al*. 2012). Sequencing data were assembled from scratch using Falcon (https://pb-falcon.readthedocs.io/en/latest/), Canu (Koren *et al*. 2017) was used to construct contigs and scaffolds, and the results were corrected in Pilon v1.18 (Walker *et al*. 2014). Finally, the benchmarking universal single-copy orthologs (BUSCO v3.0.2) tool with the Fungi odb10 data set was used to evaluate the integrity of the genome assembly (Simão *et al*. 2015).

## Genomic structure analyses

### Repeat sequences prediction

Repeat sequences were identified using the conserved model sequences of putative scattered repeats obtained in RepeatModler v1.0.4 (http://repeatmasker.org/RepeatModeler/). Then, these sequences were compared with the Swiss-Prot database (Boeckmann *et al*. 2003) for sequence alignment, and the similar protein-coding genes in this database were removed. The remaining sequences were considered as a library to search for new scattered repeat sequences using RepeatMasker v4.0.5 (http://www.repeatmasker.org/RepeatMasker/).

### Noncoding RNA analyses

Noncoding RNA predictions were conducted using tRNAscan-SE v1.3.1 (http://lowelab.ucsc.edu/tRNAscan-SE/) to predict tRNAs and RNAmmer v1.2 (https://services.healthtech.dtu.dk/services/RNAmmer-1.2/) to predict rRNAs, and the rest of noncoding RNA predictions were predicted in Rfam database (https://rfam.org/).

### Protein-coding gene prediction

To improve the accuracy of protein-coding gene predictions, Augustus v3.03 (Stanke and Morgenstern 2005), glimmerHMM v3.0.1 (Majoros *et al*. 2004), and GeneMark-ES v4.35 (Ter-Hovhannisyan *et al*. 2008) were used to predict the genomic model de novo, and Exonerate v2.2.0 (http://www.ebi.ac.uk/about/vertebrate-genomics/software/) was used to obtain the corresponding gene predictions based on the protein sequences in closely related species. At the same time, the program to assemble spliced alignments (Haas *et al*. 2003) was used to annotate gene structure and obtain the corresponding gene predictions based on transcriptome data. Finally, the prediction results derived from the above 3 methods were integrated using EVidenceModeler v2012.06.25 (Haas *et al*. 2008).

### Collinearity and ortholog analyses

*S. rugosum*, *G. lucidum*, and *G. sinense* were selected for comparative genomic analyses with *S. infundibulare*. Genome data for the former 3 species were downloaded from the Joint Genomics Institute (https://genome.jgi.doe.gov/) and the National Center for Biotechnology Information (NCBI, https://www.ncbi.nlm.nih.gov/). The genomic data of the 4 species were first formatted and analyzed using MCscan v1.2.13 (Wang *et al*. 2012), and the results were subsequently plotted. Finally, the generated graphs were edited and visually improved in Adobe Illustrator (https://www.adobe.com/cn/products/illustrator.html).

The sequences of encoded proteins were formatted, and the orthogroups for the 4 genomes were constructed using OrthoFinder v2.5.4 (Emms and Kelly 2019).

### Functional annotation of the genome

All predicted protein-coding genes of *S. infundibulare* were annotated by comparing them with various databases using Diamond v0.9.10.11 (Buchfink *et al*. 2021) and Blast+ v2.5.0 (https://blast.ncbi.nlm.nih.gov/blast/Blast.cgi). These databases were the evolutionary genealogy of genes: Non-supervised Orthologous Groups database (eggNOG, Huerta-Cepas *et al*. 2019), Gene Ontology (GO, Ashburner *et al*. 2000), the Kyoto Encyclopedia of Genes and Genomes (KEGG, Kanehisa *et al*. 2012), the Non-Redundant Protein Sequence Database (NR, https://ftp.ncbi.nlm.nih.gov/blast/db/FASTA/nr.gz), the transport classification database (TCDB, http://www.tcdb.org), and the Protein family (Pfam) databases (El-Gebali *et al*. 2019), Swiss-Prot (Boeckmann *et al*. 2003). In addition, genomic functional annotation was also performed on the Carbohydrate-Active enzymes (CAZymes) database (http://www.cazy.org/) and Antibiotics and Secondary Metabolites Analysis Shell (antiSMASH, Blin *et al*. 2019) between *S. infundibulare* and *S. rugosum*, *G. lucidum*, and *G. sinense*.

## Results and discussion

### Genome sequence and structure analyses

In the whole-genome sequencing of *S. infundibulare*, a total of 6,595,573,200- and 35,867,172,578-bp raw data were obtained using the Illumina NovaSeq and PacBio Sequel platforms. After quality control, the whole genome of *S. infundibulare* was 48,989,895 bp in length, in which the longest sequence was 4,906,148 bp and the shortest sequence was 11,236 bp (Table 1). The sequences were assembled into 88 scaffolds with a GC content of 55.84% and N50 of 3,461,589 bp. The quality of the *S. infundibulare* genome, which showed a good assembly integrity, was determined at 98.6% of complete BUSCO genes (95.4% complete and single-copy BUSCOs, 3.2% complete and duplicated BUSCOs, 1.3% missing BUSCOs, and 0.1% fragmented BUSCOs).

**Table 1.** Structural characteristics of *S. infundibulare* genome.

Size (bp)	48,989,895
GC content (%)	55.84%
Scaffold number	88
Shortest scaffold (bp)	11,236
Longest scaffold (bp)	4,906,148
N50 (bp)	3,461,589
N90 (bp)	684,864
Complete BUSCOs (%)	98.6%
Fragmented BUSCOs (%)	0.1%
Missing BUSCOs (%)	1.3%
Total interspersed repeats (bp)	4,686,040
Number of ncRNAs	145
Number of rRNAs	130
Number of tRNAs	409

Additionally, a total of 4,685,915-bp repeat sequences were found in the *S. infundibulare* genome, accounting for 9.71%. Of these, long terminal repeats accounted for the highest percentage at 3.25%, while short interspersed nuclear elements, long interspersed nuclear elements, and DNA transposons accounted for 0.00%, 0.19%, and 1.23%, respectively. A total of 145 ncRNAs, 130 rRNAs, and 409 tRNAs were also predicted in the genome (Table 1).

**Fig. 1.**
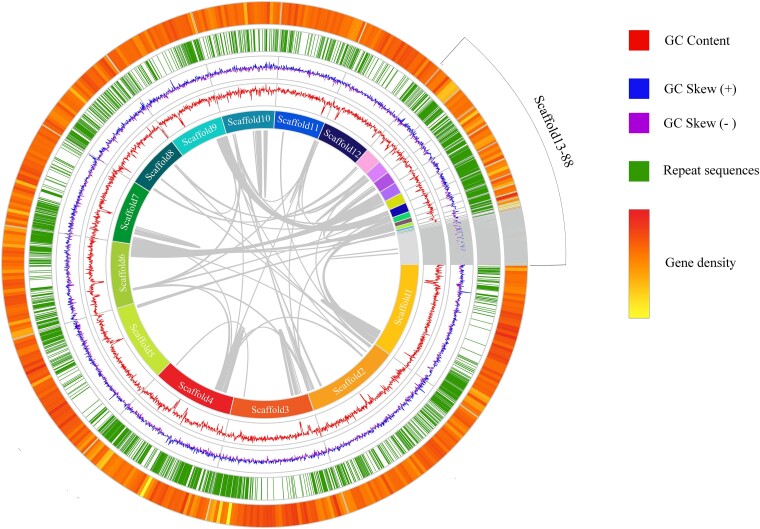
Circos of *S. infundibulare.* From inside to outside, the first circle is collinearity analyses, the second circle is a scaffold composition map, the third circle is a line chart of GC content, the fourth circle is a line chart of GC Skew, the fifth circle is a histogram of the repeat sequence, and the sixth circle is a heat map of gene density.

The collinearity analyses were performed on 88 scaffolds of *S. infundibulare*, and the basic genomic features and gene density were included in the circular genome map (Fig. 1). From the collinearity graph, it can be seen that there is no evidence for whole-genome or segmental duplications in *S. infundibulare*.

### Collinearity and ortholog analyses

The comparative analyses in genome structure were conducted among *S. infundibulare*, *S. rugosum*, *G. lucidum*, and *G. sinense* (Table 2). The genome size of 4 species ranges from 40.66 to 48.99 Mb, with the genome of *S. infundibulare* being the largest (48.99 Mb), which also had the best integrity tested via BUSCO analyses (98.6%). The GC content in the *S. infundibulare* genome (55.84%) was more similar to that in the genomes of *G. lucidum* (55.90%) and *G. sinense* (55.59%) but higher than that in the *S. rugosum* genome (50.91%). The GC content in coding sequences (CDSs) has shown a positive correlation with the length of CDSs (Wang *et al*. 2019), and it may explain the longer genome length of *S. infundibulare*. Teng *et al*. (2023) suggested that GC content may be related to the different adaptation processes of ancestors to environmental changes, while the genome-wide GC content increases faster in species with higher recombination rates per time unit (Weber *et al*. 2014). The predicted tRNA sequences of *S. infundibulare* (409) were also considerably longer than those of the other fungi.

**Table 2.** Genome comparison of *S. infundibulare*, *S. rugosum*, *G. lucidum*, and *G. sinense*

Items	*S. infundibulare*	*S. rugosum*	*G. lucidum*	*G. sinense*
Genome size (Mb)	48.99	40.66	43.30	48.96
Scaffolds	88	31	13*^a^*	69
GC content (%)	55.84%	50.91%	55.90%	55.59%
Complete BUSCOs (%)	98.6%	97.8%	95.7%	98.1%
Number of protein-coding sequences	14,146	10,181	16,113	15,688
Number of tRNAs	409	67	—	202
Source	Our study	Lin *et al*. (2021)	Chen *et al*. (2012)	Zhu *et al*. (2015)

^*a*^Chromosome level.

**Fig. 2.**
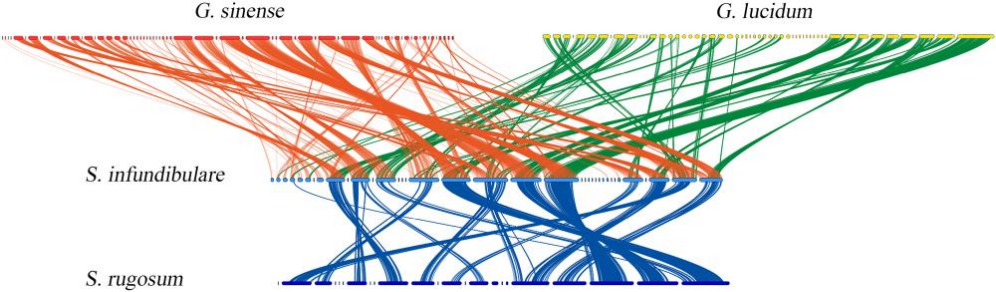
Collinearity analyses between *S. infundibulare* and *S. rugosum*, G*. lucidum*, and *G. sinense*.

The collinearity analyses between *S. infundibulare* and *S. rugosum*, *G. lucidum*, and *G. sinense* separately were conducted, and the high consistency was obtained for all three of them (Fig. 2), which can explained by the common ancestors of Ganodermataceae. This hypothesis would be in line with the inferences of previous studies (Sun *et al*. 2020). However, *S. infundibulare* showed a more concentrated collinearity with *S. rugosum*, and a more fragmented collinearity with the 2 *Ganoderma* species. This suggested that the 2 *Sanguinoderma* species may have diverged for a shorter period of time and have not yet developed more distinct characteristics.

**Fig. 3.**
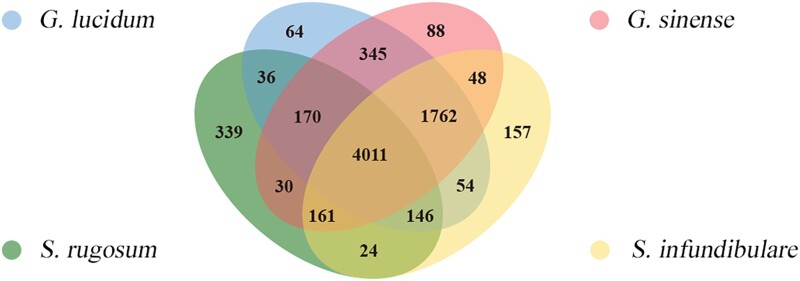
Orthologous analyses between *S. infundibulare*, *S. rugosum*, *G. lucidum*, and *G. sinense*.

Ortholog analyses were carried out for *S. infundibulare*, *S. rugosum*, *G. lucidum*, and *G. sinense* using genome sequence data (Fig. 3). A total of 4,011 orthogroups, of which 3,712 were single-copy, were selected from the 4 species. *S. infundibulare* possessed 157 specific gene families. Concurrently, the genera *Sanguinoderma* and *Ganoderma* had 24 and 345 specific gene families, respectively.

### Functional annotation

A total of 14,146 protein-coding genes were predicted in *S. infundibulare*, with an average length of 2,050.7 bp and an average of 3 exons per gene, accounting for 59.22% of the whole genome. These protein-coding genes were highly annotated in tested databases: 92.45% of them were annotated in the NR database (Supplementary Table 1), 53.27% in the Swiss-Prot database (Supplementary Table 2), and 58.23% in the Pfam database (Supplementary Table 3). In total protein-coding genes, 13,788 of them were found to relate with the CYP superfamily, while only 2,659 genes were searched after controlling *E*-value less than 1e^−5^ (Supplementary Table 4), and 1,733 protein-coding genes were annotated as transporter proteins in the TCDB (Supplementary Table 5).

### Genomic analyses of GO annotation

**Fig. 4.**
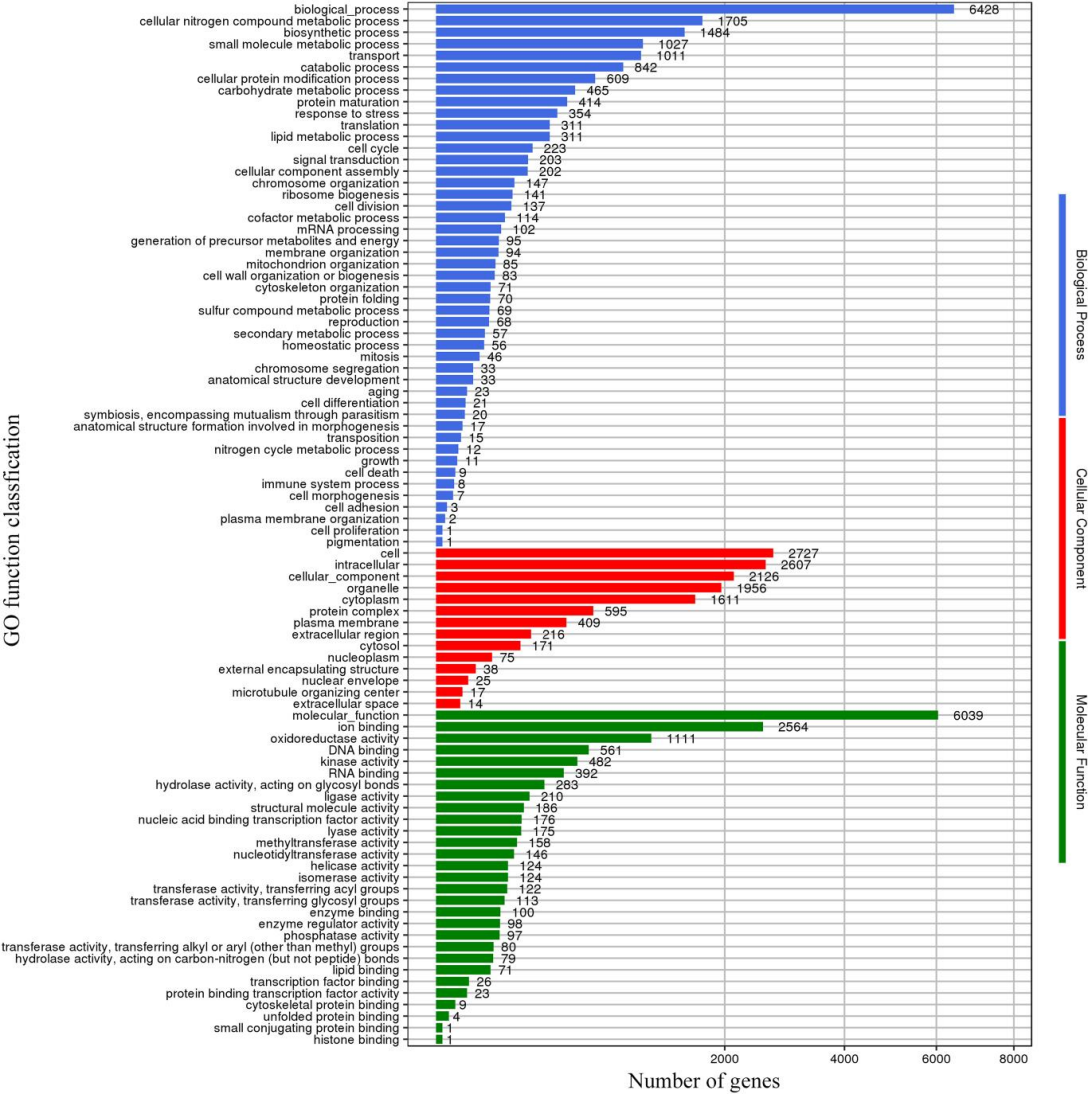
Annotation of *S. infundibulare* based on GO database.

The GO database is a widely used bioinformatic resource database that provides a standardized language for describing the functions of genes. A total of 7,147 genes, accounting for 50.52% of all protein-coding genes, were annotated in the GO database (Fig. 4). Most genes were annotated in “biological process,” i.e. biological processes (6,428), cellular nitrogen compound metabolic process (1,705), and biosynthetic processes (1,484); “cellular component,” i.e. cell (2,727), intracellular (2,607), and cellular components (2,126); and “molecular function,” i.e. molecular functions (6,039), ion binding (2,564), and oxidoreductase activity (1,111). Especially, the abundant genes associated with oxidoreductase activity terms may reveal that *S. infundibulare* had strong cellulose and lignin degradation abilities (Tian *et al*. 2021).

### Genomic analyses of KEGG annotation

**Fig. 5.**
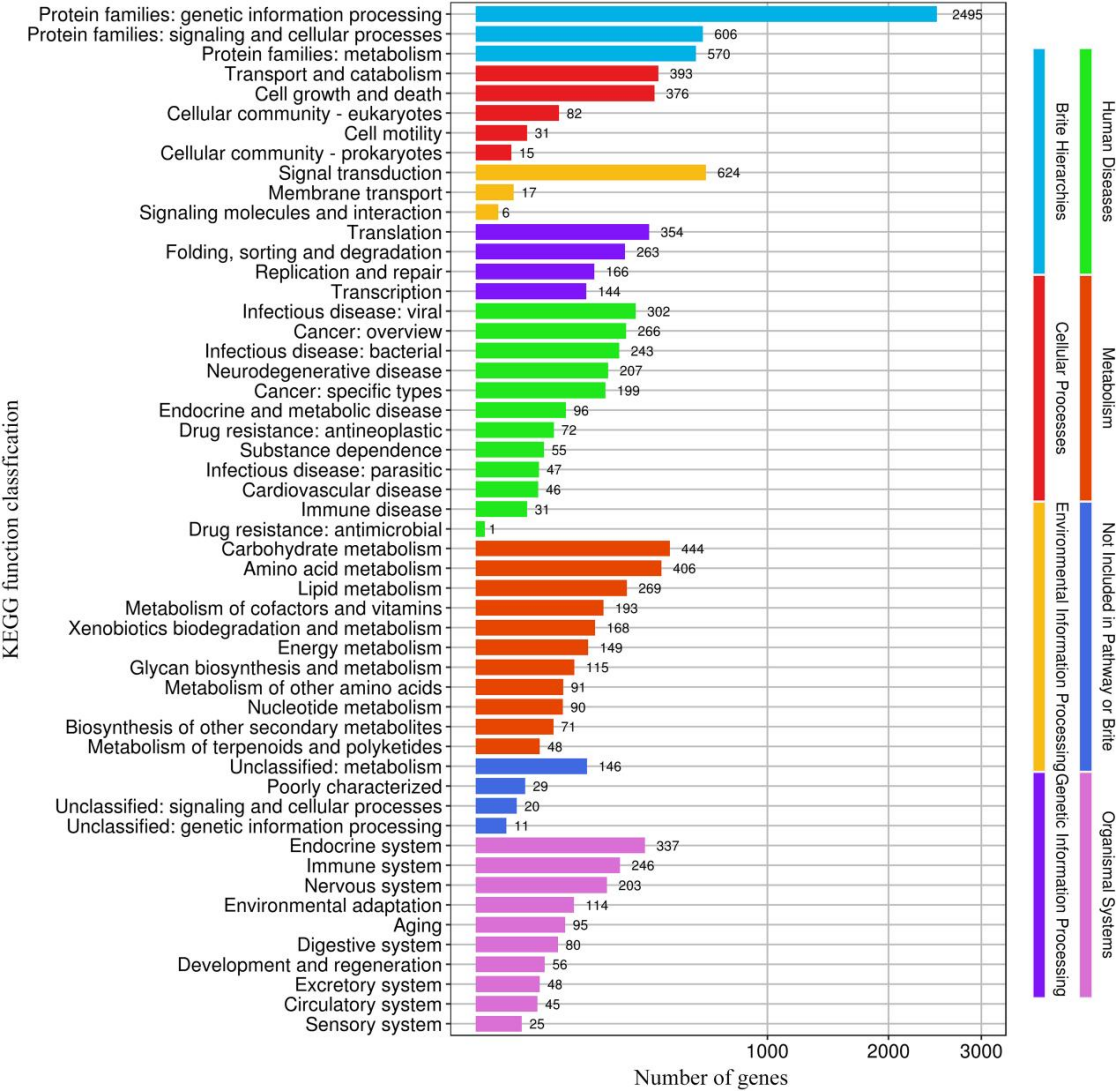
Annotation of *S. infundibulare* based on KEGG database.

**Fig. 6.**
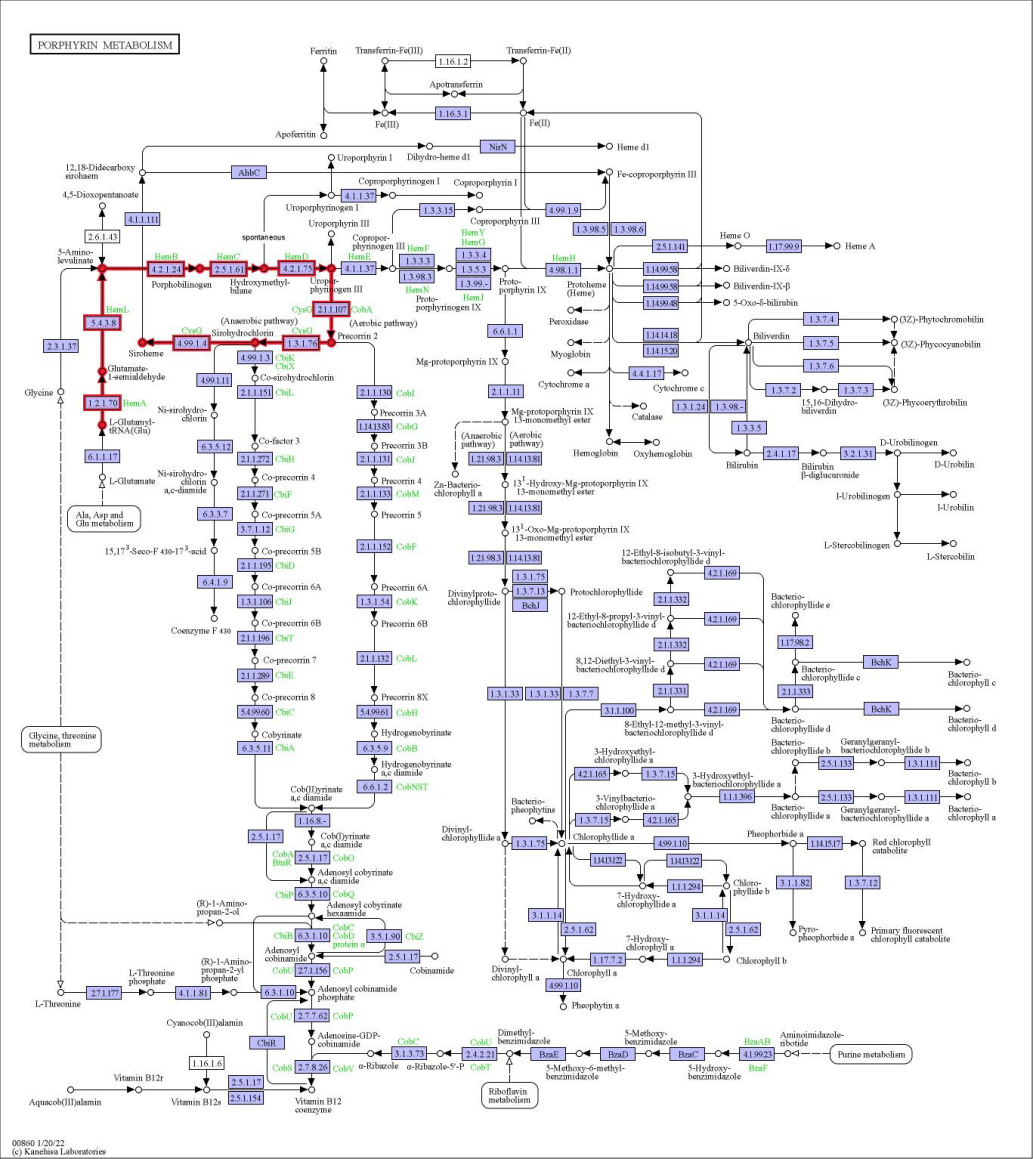
Porphyrin metabolism pathway of *S. infundibulare* (map00860) and the bold line part represent the siroheme biosynthesis pathway of *S. infundibulare*.

KEGG database annotations play an important role in understanding the detailed function of genes involved in biological systems, especially the category corresponding to metabolic pathways. In the KEGG database, 3,856 genes of *S. infundibulare* accounted for 27.26% of the whole genome, which were annotated in 8 physiological processes. In the second layer of KEGG pathway terms, the main protein-coding genes were annotated in genetic information processing (2,495), signal transduction (624), signaling and cellular processes (606), and metabolism pathways (570, Fig. 5). Besides, there is a specific gene family of *Sanguinoderma* that is included in the siroheme biosynthesis pathway. It is associated with the synthesis of compounds that can produce a blood red color (Spencer *et al*. 1993). Based on previous research, siroheme has been found in *Saccharomyces cerevisiae* and is related to the nicotinamide adenine dinucleotide-binding process (Schubert *et al*. 2002), which is consistent with our annotation in the Pfam database. In KEGG pathways, siroheme biosynthesis of *S. infundibulare* was a part of the porphyrin metabolism pathway (Fig. 6). Porphyrins belong to a kind of macrocyclic compound that was applied in cancer diagnosis and treatment (Chen, Zhu, *et al.* 2021). The porphyrin metabolism pathway can generate heme and siroheme. Dailey *et al*. (2017) found that this pathway is the most ancient, and it can convert siroheme to protoheme in Archaea without oxygen. Siroheme is a type of heme cofactor that has been shown to be involved in the formation of ammonia and sulfides in microorganisms and plants (Raux *et al*. 2003). It is a synthetic product of uroporphyrinogen III, generated after 2 steps of methylation, 1 step of dehydrogenation, and 1 step of chelation reaction (Zhu *et al*. 2020).

### Genomic analyses of CAZymes annotation

**Fig. 7.**
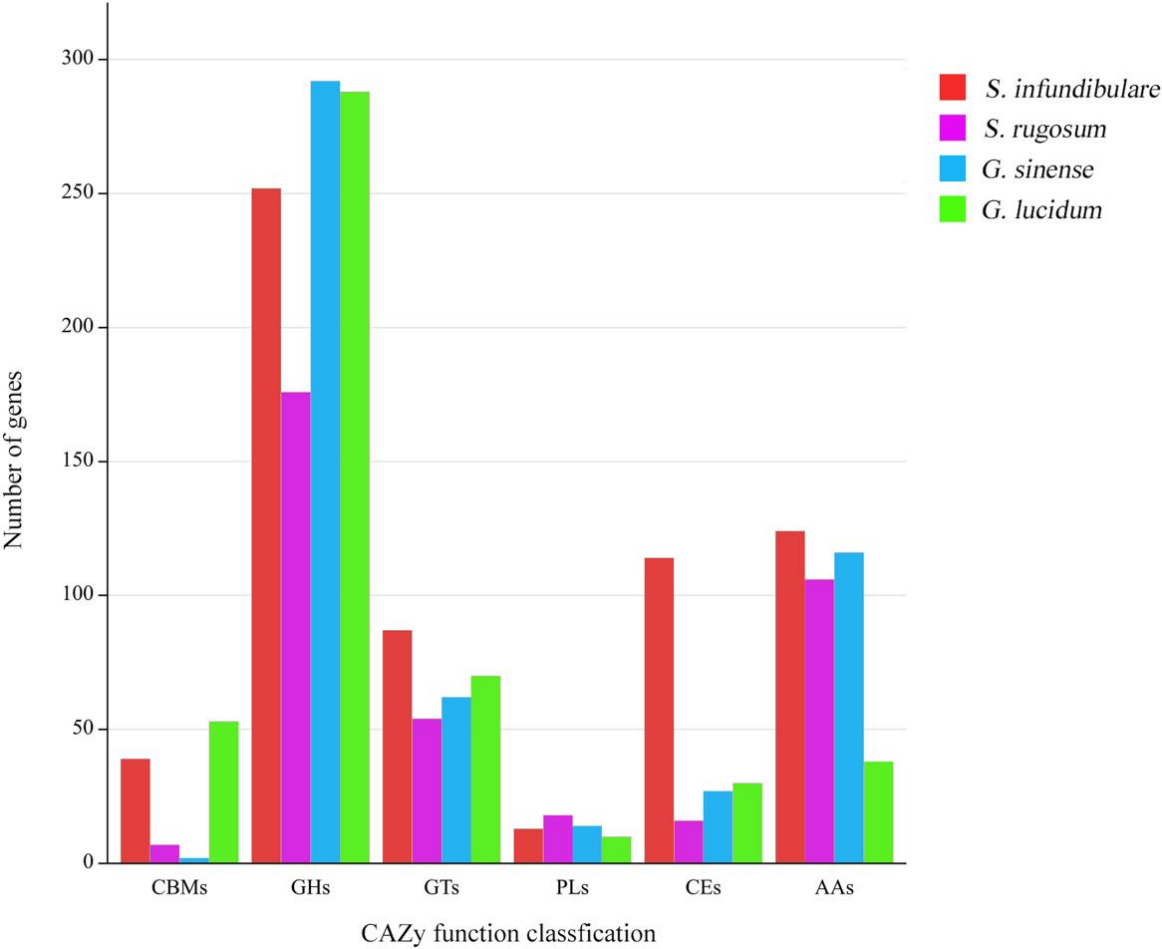
Annotation of *S. infundibulare*, *S. rugosum*, *G. lucidum*, and *G. sinense* based on CAZymes database.

Performing CAZymes database annotation in *S. infundibulare* helps identify and classify the protein-coding genes related to carbohydrate-active enzymes, which enables a better understanding of its functions and roles. A total of 629, 377, 513, and 489 protein-coding genes in *S. infundibulare*, *S. rugosum*, *G. lucidum*, and *G. sinense*, respectively, were annotated in the CAZymes database. The genomes of 4 species were dominated by the gene families of glycoside hydrolases (GH) and glycosyltransferases (GT). *S. infundibulare* and *G. lucidum* presented plentiful gene families of carbohydrate-binding module (CBM) than the other 2 species, and the former was mostly annotated with gene families of carbohydrate esterases (CEs, Fig. 7). The gene families of CE10 (62), CE16 (23), and CE1 (15) were detected in the genome of *S. infundibulare*. The gene families of CE10 were contained in aromatase, carboxylesterase, and acetylcholinesterase activities (de Vries and de Vries *2*020), CE16 was contained in acetylesterase activity (Koutaniemi *et al*. 2013), and CE1 was contained in acetyl xylan esterase, cinnamic acid esterase, and ferulic acid esterase activities (Li *et al*. 2020). The past studies have shown that acetylesterase, acetyl xylan esterase, and cinnamic acid esterase contribute to the degradation of cellulose and hemicellulose (Tsujiyama and Ueno 2011; Yang *et al*. 2013; Mai-Gisondi *et al*. 2015). It suggested that *S. infundibulare* may be more active in the degradation of cellulose and hemicellulose. Moreover, the abundant gene families of CBM were detected in *S. infundibulare* (39) and *G. lucidum* (53), which can enhance the hydrolysis activity of cellulase (Byrt *et al*. 2012).

The orthogroups found in *S. infundibulare*, *S. rugosum*, *G. lucidum*, and *G. sinense* were also annotated in the CAZymes database. Most orthogroups were annotated as GH and GT families, of which the most abundant were GH3, GH18, and GT2 (Supplementary Table 6). GH3 and GH18 are related to β-glucosidase, xylan 1,4-beta-xylosidase, and β-glucosylceramidase activities (Faure 2002), and the GT2 is related to cellulose synthase and chitin synthase activities (Breton *et al*. 2006), chitinase, lysosomal, and acetylamino glucosidase activities (Lienemann *et al*. 2009). Like most white-rot fungi, the CAZymes in *S. infundibulare* have outstanding lignocellulosic degradation ability, which may be due to the common saprophytic habit (Větrovský *et al*. 2013; Sista Kameshwar and Qin 2018). The specific gene families in *Ganoderma* were annotated with 5 GH18, while the specific gene families in *Sanguinoderma* were annotated with 2 auxiliary activities 9 and 2 CBM1, which were considered as typical cellulose decomposing enzyme families of white-rot fungi (Levasseur *et al*. 2014).

### Genomic analyses of antiSMASH annotation

Conducting antiSMASH database annotation is essential for identifying and predicting biosynthetic gene clusters in fungal genomes, which in turn allows for the exploration and discovery of a wide range of biologically active compounds and potential drug molecules. In the antiSMASH database, 33, 36, 32, and 36 secondary metabolic gene clusters were annotated for *S. infundibulare*, *S. rugosum*, *G. lucidum*, and *G. sinense*, respectively (Supplementary Table 7). All 4 species have annotated a large number of terpene gene clusters and nonribosomal peptide synthetase (NRPS)-related gene clusters, among which 18 terpenes and 10 NRPS-related gene clusters (including 1 NRPS gene clusters and 9 NRPS-like gene clusters) have been annotated in *S. infundibulare*. Many studies have pointed out that fungi in Ganodermataceae contain a large amount of terpenoids, which were related to their medicinal value in anti-inflammatory and anticancer effects (Tang *et al*. 2010; Baby *et al*. 2015; Galappaththi *et al*. 2022), and some studies have pointed out that nonribosomal peptides catalyzed by NRPS also have anti-inflammatory and antitumor effects (Nikolouli and Mossialos 2012; Süssmuth and Mainz 2017; Iacovelli *et al.* 2021).

### Genomic analyses of eggNOG annotation

**Fig. 8.**
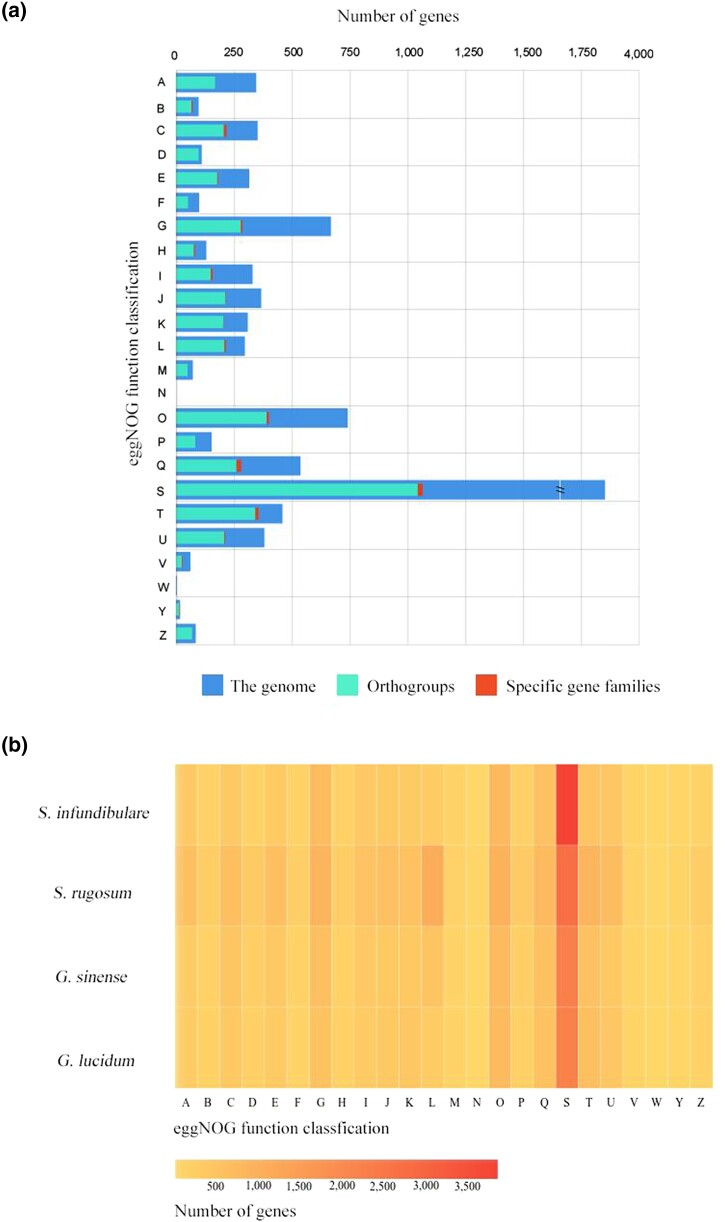
a) Annotation of the genome and specific gene families of *S. infundibulare* and orthologroups of 4 species based on eggNOG database. b) Annotation of *S. infundibulare*, *S. rugosum*, *G. lucidum*, and *G. sinense* based on eggNOG database. Category functions: (A) RNA processing and modification; (B) chromatin structure and dynamics; (C) energy production and conversion; (D) cell cycle control, cell division, chromosome partitioning; (E) amino acid transport and metabolism; (F) nucleotide transport and metabolism; (G) carbohydrate transport and metabolism; (H) coenzyme transport and metabolism; (I) lipid transport and metabolism; (J) translation, ribosomal structure, and biogenesis; (K) transcription; (L) replication, recombination, and repair; (M) cell wall/membrane/envelope biogenesis; (N) cell motility; (O) posttranslational modification, protein turnover, and chaperones; (P) inorganic ion transport and metabolism; (Q) secondary metabolites biosynthesis, transport, and catabolism; (R) general function prediction only; (S) function unknown; (T) signal transduction mechanisms; (U) intracellular trafficking, secretion, and vesicular transport; (V) defense mechanisms; (W) extracellular structures; (Y) nuclear structure; and (Z) cytoskeleton.

The eggNOG database collects a large number of functional proteins from other organisms, and it can annotate the function of genes based on blasting similar sequences in the database. *S. infundibulare* presented 9,891 annotated genes in the eggNOG database (Supplementary Table 8), excluding the S (function unknown) functions, predominantly associated with O (posttranslational modification, protein turnover, and chaperones), G (carbohydrate transport and metabolism), and Q (secondary metabolites biosynthesis, transport, and catabolism) functions. The gene with O, G, and Q functions should be related to the secondary and primary metabolism of white-rot fungi on wooden substrates (Zhao *et al*. 2019). However, the annotation results of *S. infundibulare* were not completely consistent with previous study on *G. lucidum* (Yu *et al*. 2015). Both of these focus on the G function, but in other aspects, *G. lucidum* placed more emphasis on the E (amino acid transport and metabolism) and C (energy production and conversion) functions. The most annotated functions in the orthogroups of the 4 species, except for the S function, were the O, T (signal transduction mechanisms), and G functions. In addition, Q functions were the most annotated in the specific gene families of *S. infundibulare* (Fig. 8a). No significant differences were detected among the 4 Ganodermataceae species, except that *S. infundibulare* had more S functions (Fig. 8b). Compared with the genus *Ganoderma*, the specific gene families of *Sanguinoderma* had additional H (coenzyme transport and metabolism) functions associated with siroheme synthase, which corresponded to the annotation results of the KEGG database mentioned above.

## Conclusion

This study provided the whole-genome sequencing data and basic genomic features of *S. infundibulare*, compared it with the genomes of *S. rugosum*, *G. lucidum*, and *G. sinense* for comparative genomic analysis, and revealed the potential functions of 4 species. *S. infundibulare* has a relatively large genome with high quality in Ganodermataceae, and it has more concentrated collinearity with *S. rugosum*. Based on the annotation of 14,146 protein-coding genes of *S. infundibulare*, it was found that *S. infundibulare* has high annotation rates in tested databases. In the genus *Sanguinoderma*, there is a specific gene family related to siroheme biosynthesis, and it may be associated with the color of injured pore surface that can change to blood red in this genus. In conclusion, this study contributes to enriching the genetic database for the family Ganodermataceae, further understanding its genomic structure and function and also providing a solid foundation for the further development and utilization of the genus *Sanguinoderma* in the future.

## Supplementary Material

jkae005_Supplementary_Data

## Data Availability

The raw data of *S. infundibulare* have been deposited in NCBI under the BioProject number PRJNA994897, and the accession number was JAVLRN000000000. The annotations of genome are available at Figshare (http:/doi.org/10.6084/m9.figshare.24886239). Supplemental material available at G3 online.
